# 

**Published:** 1894-05

**Authors:** 


### CONTENTS:

Article I.—A New Practical Method of Fusing Platinum. By L E. Custer, B. S., D. D. S. - - - - - - 1
" II.—Saving the Teeth. By W. Irving Thayer, D. D. S., M. D. - - - - - - - - 4
" III.—Manipulative Ability. - - - - - - - 7
" IV.—Antiseptic Dentistry. By Garrett Newkirk, M. D. - 8
" V.—Storage Batteries and Their Uses in Dentistry. By W. W. Vance, D. D. S. - - - - - 19
" VI.—Retention of Artificial Dentures. - - - - 25
" VII.—The Physiology of Conception. By Dr. A. D. Barr. 29
" VIII.—The Cracking of Teeth in Soldering. By L. P. Haskell, D. D. S. - - - - - - 33

### COMMUNICATED.

The American Dental Society. - - - - - - 35
Illinois State Dental Society. - - - - - - 35

### EDITORIAL, Etc.

Dentists of Maryland and Washington Meet. - - - - - 36
The Medical Law of Maryland. - - - - - - 37
Love and Law. - - - - - - - - 41

### MONTHLY SUMMARY.

Spongy Gums. - - - - - - - - 43

How an Old Story Originated. - - - - - - 44

Electricity in 1900. - - - - - - - - 45

Trichloracetic Acid. - - - - - - - - 45

Effects of Pregnancy. - - - - - - - 46

Putrescent Pulps. - - - - - - - - 47

Health and Happiness. - - - - - - - 47

Bacteriology. - - - - - - - - - 48

To Cut Off Crowns. - - - - - - - - 48

Testing Gold and Silver. - - - - - - - 48

